# Revealing Cues for Fungal Interplay in the Plant–Air Interface in Vineyards

**DOI:** 10.3389/fpls.2019.00922

**Published:** 2019-07-25

**Authors:** Ahmed Abdelfattah, Simona M. Sanzani, Michael Wisniewski, Gabriele Berg, Santa O. Cacciola, Leonardo Schena

**Affiliations:** ^1^Dipartimento di Agraria, Università Mediterranea di Reggio Calabria, Reggio Calabria, Italy; ^2^Department of Ecology, Environment and Plant Sciences, Stockholm University, Stockholm, Sweden; ^3^Dipartimento di Scienze del Suolo, della Pianta e degli Alimenti, Università degli Studi di Bari Aldo Moro, Bari, Italy; ^4^U.S. Department of Agriculture–Agricultural Research Service (USDA-ARS), Kearneysville, WV, United States; ^5^Institute of Environmental Biotechnology, Graz University of Technology, Graz, Austria; ^6^Dipartimento di Agricoltura, Alimentazione e Ambiente, Università degli Studi di Catania, Catania, Italy

**Keywords:** grapes, microbiota, fungal community, aerobiology, spore trap, ITS amplicon libraries, holobiont, metagenomics

## Abstract

Plant-associated microorganisms play a crucial role in plant health and productivity. Belowground microbial diversity is widely reported as a major factor in determining the composition of the plant microbiome. In contrast, much less is known about the role of the atmosphere in relation to the plant microbiome. The current study examined the hypothesis that the atmospheric microbiome influences the composition of fungal communities of the aboveground organs (flowers, fruit, and leaves) of table grape and *vice versa*. The atmosphere surrounding grape plantings exhibited a significantly higher level of fungal diversity relative to the nearby plant organs and shared a higher number of phylotypes (5,536 OTUs, 40.3%) with the plant than between organs of the same plant. Using a Bayesian source tracking approach, plant organs were determined to be the major source of the atmospheric fungal community (92%). In contrast, airborne microbiota had only a minor contribution to the grape microbiome, representing the source of 15, 4, and 35% of the fungal communities of leaves, flowers, and fruits, respectively. Moreover, data indicate that plant organs and the surrounding atmosphere shared a fraction of each other’s fungal communities, and this shared pool of fungal taxa serves as a two-way reservoir of microorganisms. Microbial association analysis highlighted more positive than negative interactions between fungal phylotypes. Positive interactions were more common within the same environment, while negative interactions appeared to occur more frequently between different environments, i.e., atmosphere, leaf, flower, and fruit. The current study revealed the interplay between the fungal communities of the grape phyllosphere with the surrounding air. Plants were identified as a major source of recruitment for the atmospheric microbiome, while the surrounding atmosphere contributed only a small fraction of the plant fungal community. The results of the study suggested that the plant–air interface modulates the plant recruitment of atmospheric fungi, taking a step forward in understanding the plant holobiont assembly and how the atmosphere surrounding plants plays a role in this process. The impact of plants on the atmospheric microbiota has several biological and epidemiological implications for plants and humans.

## Introduction

Microorganisms play a major role in shaping ecosystems, contributing to nutrient cycling, primary production, litter decomposition, and multitrophic interactions (Mahnert et al., 2015; Abdelfattah et al., 2018). In recent years, the importance of the functional role that plant-associated microorganisms play in plant health, productivity, and environmental resilience has been increasingly recognized (Berendsen et al., 2012; Berg et al., 2014a). The beneficial effects of the microbiota on plant health include increased tolerance to biotic and abiotic stresses (Eleonora et al., 2015; Unyarat et al., 2016), growth promotion, and increased nutrient intake (van der Heijden et al., 2015). In recognition of these effects, the hologenome theory suggests that the holobiont (host plus symbionts) with its hologenome (host genome plus microbiome) act as a single evolutionary unit (Zilber-Rosenberg and Rosenberg, 2008). In this regard, the source, dissemination, spatial distribution, and conservation of the plant microbiome are critical to understanding the plant holobiont. Although soil is often reported to be a major source of plant microbiota (Bulgarelli et al., 2013; Edwards et al., 2015; Belda et al., 2017; Hassani et al., 2018), the role of the atmosphere as a source for the recruitment and dissemination of the plant microbiota is poorly understood.

The atmosphere (air) has long been considered a poor ecosystem for the growth and multiplication of microbes, but recent studies, especially of indoor, artificial environments, have demonstrated the presence of a substantial number of microorganisms in air samples (Elbert et al., 2007; Fröhlich-Nowoisky et al., 2009; Mahnert et al., 2015). These atmospheric microorganisms have been implicated in metabolizing atmospheric organic matter, influencing the earth’s biogeochemical cycles, affecting atmospheric chemistry, precipitation cycles, and even climate change (Delort et al., 2010; Rosenfeld et al., 2014; Behzad et al., 2015). Among airborne microorganisms, fungi have a particular importance when it comes to plants, since major plant pathogens are dispersed by air currents.

The microbiome of grapevine (*Vitis vinifera* L., 1753) has been extensively studied. It comprises approximately 10^3^ and 10^5^ colony-forming units (cfu) of fungi and bacteria per gram of fresh tissue, respectively (Verginer et al., 2010; Pinto et al., 2014; Marasco et al., 2018). The importance of microorganisms associated with grape plants in vineyards has been progressively recognized due to their impact on plant health and productivity (Grube et al., 2011; Schmid et al., 2011; Berg et al., 2014a). The composition of the grape microbiome was also found to distinguish different viticultural regions and contribute to determining wine properties (Bokulich et al., 2016). While soil is often reported to be a major source of grapevine microbiota (Belda et al., 2017; Hassani et al., 2018), the impact of the surrounding atmosphere as a source of recruitment is completely unknown. The hypothesis examined in the present study is that the atmospheric microbiome influences the composition of the fungal communities associated with the aboveground organs (flowers, fruit, and leaves) of table grapes and *vice versa*. Therefore, the fungal microbiota associated with plant organs (flowers, leaves, and fruits) and the surrounding air were investigated in order to evaluate their reciprocal role in the modulation of fungal communities.

## Materials and Methods

### Vineyards

Table grape vineyards, variety “Italia” grafted on Kober 5BB (Berlandieri × Riparia), with plantation of the same age (10 years old) were used in the present study. Both vineyards were located in Castellaneta, Province of Taranto, Italy (40°34′35.1″N 16°56′27.4″E and 40°33′35.0″N 16°55′47.3″E, respectively). The vineyards were approximately 2 km distant from each other and situated at the same altitude (approximately 70 m a.s.l). The grapevines were trained with the “tendone” system, which favors horizontal growth of the canopy at around 2 m from the ground.

### Sample Collection

Samples were collected from vineyards, located in Castellaneta, Province of Taranto, Italy (40°34′35.1″N 16°56′27.4″E and 40°33′35.0″N 16°55′47.3″E), in three uniform plots consisting of 10 plants. Plots were approximately 150 m apart. To collect airborne fungal spores, specific traps containing a thin layer (1 ml) of liquid Vaseline containing phenol were prepared with standard Petri dishes (100 mm × 15 mm) without lid. Traps were hung using iron wires, and suspended 20 cm below the main vegetation layer, which was approximately 150 cm above the soil surface. Each sampling plot consisted of 10 spore traps (one per plant), making a total of 30 traps per vineyard. Spore traps were left for 1 week at the beginning of each month, starting from flowering (first half of June), and continued monthly until harvest (first half of October), resulting in five sampling time points and an overall total of 300 spore traps. Flowers, fruit, and leaves were sampled from the same plots, simultaneously to traps. Ten leaves and fruit bunches were collected from each pot (one per plant). These samples were pooled to have three biological replicates per each sampling time and vineyard (*n* = 90).

### DNA Extraction

All collected samples were transported to the lab in cool containers (5°C). Leaves and bunches where frozen, lyophilized (Labconco^®^ FreeZone 2.5), and grounded in liquid nitrogen with sterile mortars and pestles. Total DNA was extracted from 80 mg of homogenate tissues using DNeasy Plant Mini Kit (Qiagen^®^). Concentration and quality of extracted DNA were assessed using Nanodrop spectrophotometer (Thermo Fisher Scientific, Inc., United States). All samples were diluted to have a uniform DNA concentration of 10 ng μl^-1^.

To extract DNA from spore traps, 1.5 ml of a preheated (65°C) buffer, containing 2% cetyltrimethylammonium bromide (CTAB), 1.4 M NaCl, 20 mM EDTA, pH 8, 100 mM Tris–HCl, pH 8, and 1% PVP, was added to each plate and the surface was scrubbed with a sterile L-shaped plastic rod in order to detach fungal spores. The resulting suspension obtained from each plate was used for total DNA using the protocol described by Schena and Cooke (2006). Extracts were purified with the Agencourt AMPure XP system (Beckman Coulter, Inc.) to eliminate any phenol or traces of Vaseline. Purified DNA extracts from plates of each plot (10 subsamples) were pooled together to have three biological replicates per each sampling time and vineyard. Pooled samples were analyzed using a Nanodrop spectrophotometer (Thermo Fisher Scientific, Inc., Waltham, MA, United States) and DNA concentration was adjusted to a uniform concentration of 10 ng/μl.

### Amplicon Generation and Sequencing

The universal primers ITS3_KYO2 and ITS4 were used to amplify the ITS2 region of the ribosomal DNA (Toju et al., 2012). Both primers were modified to include Illumina adaptors^1^ for subsequent multiplexing. PCR reactions were conducted in a total volume of 25 μl containing 12.5 μl of KAPA HiFi HotStart ReadyMix (Kapa Biosystems, Wilmington, MA, United States), 1.5 μl of each primer (10 μM), and 2.5 μl of DNA template. Reactions were incubated in a T100 thermal cycler (Bio-Rad, Hercules, CA, United States) for 3 min at 98°C, followed by 30 cycles of 30 s at 95°C, 30 s at 50°C, and 30 s at 72°C. All reaction cycles ended with a final extension time of 1 min at 72°C. A negative control in which nuclease-free water (QIAGEN, Valencia, CA, United States) replaced template DNA was included at all of the assessment times. All amplicons, including amplification mixtures from negative controls were sequenced using Illumina MiSeq V3 (2 × 300 bp) chemistry according to the manufacturer’s instructions.

### Data Analysis

Illumina adaptors were clipped and low-quality reads were removed by Trimmomatic 0.36 (Bolger et al., 2014) using a sliding window trimming, cutting once the average quality within the window of four bases falls below a quality threshold of 15. Paired-end reads were then merged utilizing PANDAseq with default parameters and read overlap of 20 bp (Masella et al., 2012). Chimeric sequences were identified and removed using VSEARCH 1.4.0 (Rognes et al., 2016). UCLUST algorithm (Edgar, 2010), as implemented in QIIME 1.9.1 (Caporaso et al., 2010), was used to cluster sequences queried against the UNITE dynamic database released on 01.12.2017 (Abarenkov et al., 2010) at a similarity threshold of 97%. Sequences that failed to cluster against the database were *de novo* clustered using the same algorithm. After removing singletons, the most abundant sequences in each Operational Taxonomic Unit (OTU) were selected as representative sequences and used for the taxonomic assignment using the BLAST algorithm (Altschul et al., 1990) as implemented in QIIME 1.9.1.

### Diversity Metrics and Statistics

Rarefaction to an even sequencing depth of 1,000 reads per sample was used to normalize the OTU table. The rarefied OTU table was used to calculate alpha diversity indices including Observed Species (*Sobs*) and Shannon metrics. Non-parametric two-sample *t* test was used to compare alpha diversities. MetagenomeSeq’s cumulative sum scaling (CSS) (Paulson et al., 2013) was used as a normalization method for other downstream analyses. The CSS normalized OTUs table was analyzed using Bray–Curtis metrics (Bray and Curtis, 1957) and utilized to evaluate beta diversity and construct PCoA plots using Emperor (Lozupone and Knight, 2005). Similarity in community composition was tested *via* ANOSIM in QIIME 1.9.1 using 999 permutations. The most prevalent taxa (≥0.1%) were selected in order to evaluate the significance of differences in the relative abundance of the detected taxa using Kruskal–Wallis method (Kruskal and Wallis, 1952). Significance in all of the analyses was determined using 999 Monte Carlo permutations, and Benjamini–Hochberg (FDR) corrections were used to adjust the calculated *p* values. Cytoscape 3.3.0 was used to analyze the CSS normalized OTU table and construct network figures to visually display unique and shared OTUs between samples.

### Source Tracking

To estimate the source of the fungal community present in air as well as leaf, fruit, and flower (designated here environments), we used SourceTracker2^2^. This tool was designed to estimate proportion/fraction of community that originates from a set of source environments by using a Bayesian approach and Gibb’s sampling method (Knights et al., 2011). Here, we tested each environment for being a source or a sink. For this purpose, we conducted multiple runs by setting one environment as sink while setting the others as sources and repeated this process for all environments. To estimate the fraction of communities that is shared between source and sink, the following assumptions were made: if *x* is the fraction of environment *A* that originate from environment *B*, and *y* is the fraction of environment *B* that originate from environment *A*, then *z* (fraction of fraction), calculated as (*x* ^∗^ *y*) represents the fraction of communities that return from *A* to *B* and/or *B* to *A*. Furthermore, the sum of *z* obtained from all sources in a given environment represents the total fraction of a community that return to the same environment. Hence, the sum of *z* can be considered as an index of conservation; higher values indicate a stable community and lower values indicate variable communities.

### Interaction Network

Inferred fungal associations (co-occurrence and mutual exclusion) within grape and air samples were computed using the CoNet (v1.1.1. beta) plugin within Cytoscape (v3.6.1). Rare taxa were discarded from the analysis by considering only OTUs present in at least 20 samples. The associations/interaction between fungal phylotypes were calculated using a combination of five methods, i.e., Spearman, Pearson coefficients, Bray–Curtis and Kullback–Leibler dissimilarity metrics, and Mutual Information (Kullback and Leibler, 1951; Bray and Curtis, 1957). *p* values for each metric were calculated by 100 permutation using edgeScores routine and shuffle rows as resampling strategy. This was followed by bootstrapping step using 100 iterations, where unstable edges were removed. All the calculated method-specific *p* values of an edge were merged into one *p* value using Brown’s method (Brown, 1975), and Benjamini–Hochberg multiple testing correction was used false-discovery rate correction (Benjamini and Hochberg, 1995). The created network was clustered using simple division based on connectivity, based on Connected Components algorithm as implemented in clusterMaker2 (Morris et al., 2011).

## Results

### OTUs Distribution and Shared Communities

**FIGURE 1:**
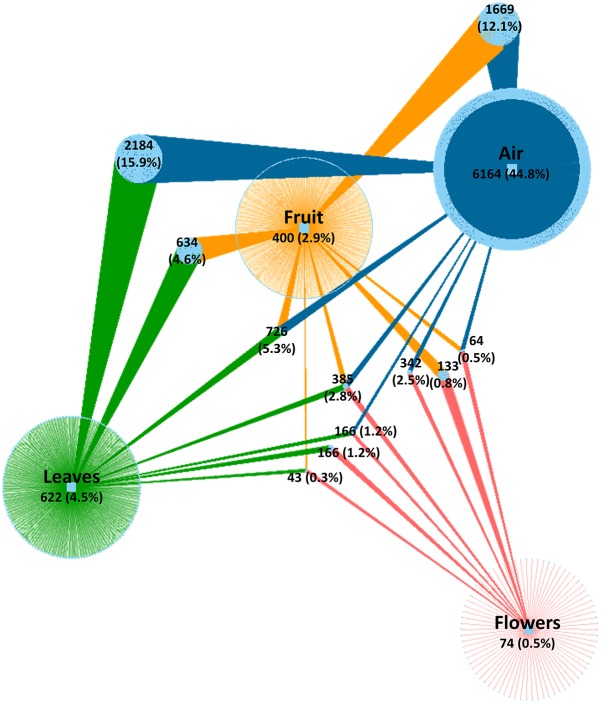
Network of shared and unique fungal OTUs among investigated samples. Colored edges (links) represent OTUs associated to sample types (air, fruit, leaves, and flowers). Light blue circle nodes connecting two or more samples represent shared OTUs. Numerical values indicate the number of OTUs and their percentage relative to the total number of OTUs.

After quality evaluation and deletion of chimeric reads, singletons, and plant sequences, a total of 5,566,210 fungal sequences were obtained from the 90 analyzed samples (air, leaves, and flowers/fruits) collected monthly (from June to October) from table grape vineyards. The sequences were assigned to 13,773 OTUs using a 97% similarity threshold (Supplementary Table S1). The number of OTUs varied significantly between air samples and grape organs. The highest number of OTUs was present in air samples (11,700 OTUs) followed by leaves (4,926 OTUs), fruit (4,011 OTUs), and flowers (1,373 OTUs). The number of unique OTUs followed the same order, accounting for 6,164, 622, and 74 OTUs, respectively. The largest fraction of shared OTUs (5,536 OTUs, 40.3%) was between air samples and the various grape organs. Overall, atmospheric samples shared 3,461, 2,844, and 957 OTUs with leaves, fruits, and flowers, respectively, of which 2,184, 1,669, and 342 OTUs were exclusively shared between air and each of these organs, respectively (Figure 1). Notably, 385 OTUs (2.8%) were common to all samples and only 43 OTUs (0.3%) were shared between grape organs (leaves, fruit, and flowers).

### Source and Dissemination of the Fungal Communities

**FIGURE 2:**
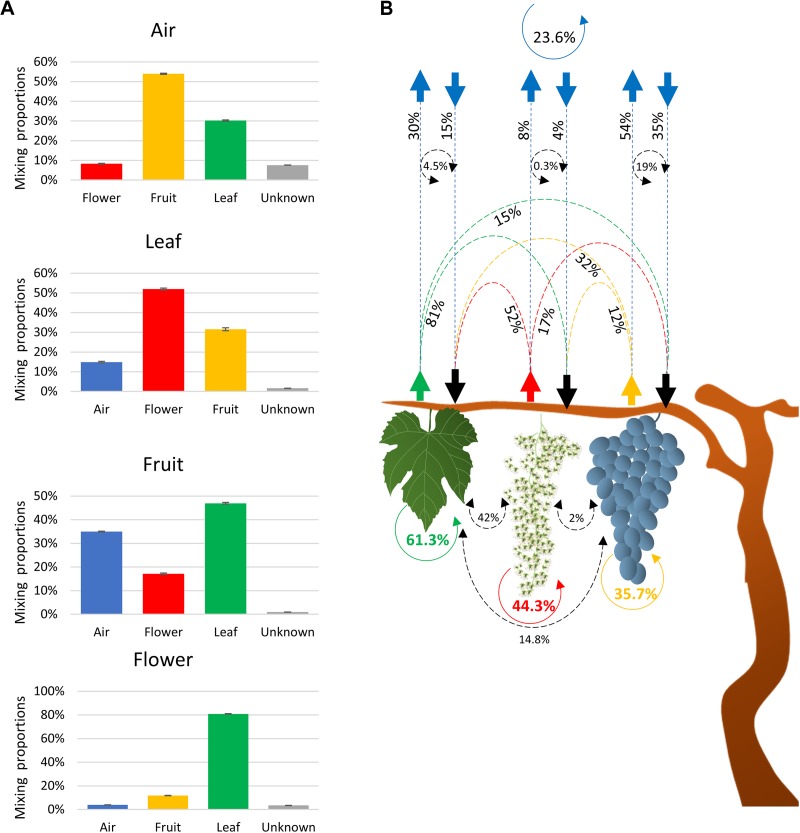
Charts showing fractions of the estimated sources of fungal communities in each sink environment (air, leaves, and flowers/fruit) **(A)** and schematic description of the estimated source of the fungal communities associated with air and plant organs **(B)**. The scheme shows the estimated movement of communities originated from leaves (green dashed arrows), flower (red dashed arrows), fruit (orange dashed arrows), and air (blue arrows). Dashed black circle arrows indicate the percentages of community commonly exchanged between two environments. Solid colored circle arrows showed the percentages of community that retune to the same environment from all the other sources **(B)**.

SourceTracker2 software was used to determine the source of the fungal community (Knights et al., 2011). Results indicated that fungi associated with grape organs (leaf, flower, and fruit) mainly originated from the plant itself (Table 1 and Figure 2A,B). The leaf community originated from flower (52%), fruit (32%), and atmospheric (15%) communities. Fungi associated with flowers were primarily recruited from leaves (81%), fruit (12%), and air (4%). Furthermore, the fruit community originated from leaves (47%), flowers (17%), and air (35%). In contrast, most of the atmospheric community (92%) originated from plant tissues, i.e., fruit (54%), leaves (30%), and flowers (8%), while the remaining 8% was from an unknown source.

To test whether the communities associated with each environment could return to the same environment, the plant contribution to the atmospheric community was multiplied by the corresponding contribution of the atmospheric microbiota to that plant fungal community. Based on these calculations, the fungal taxa that may have originated from fruit, leaves, and flowers and returned to the same organs through the air was estimated to be 19, 4.5, and 0.3%, respectively (Figure 2B). Similarly, 14.8 and 2.0% of the leaf and flower fungal taxa were estimated to contribute to the fruit fungal community and may have originated from the fruit itself. Lastly, 42% of leaf fungal taxa contributed to the flower fungal community and may have originated from the flowers themselves. These estimates were considered as factions of communities that return to the same organ and that were common to both sinks and sources. Therefore, the sum of these fractions represents the overall fraction of a community associated with an environment (plant organ or air) that may have returned to the same environment from different sources. The sum of fractions indicated that 23.7, 35.7, 61.3, and 44.3% of air, fruit, leaf, and flower communities, respectively, returned to the same organs and were interchangeable with all of the other environments.

**Table 1:** Estimated source of fungal communities associated to air, fruit, leaves, and flowers (read data according to the first column).

	Air	Fruit	Leaf	Flower	Unknown
Air	–	54%	30%	8%	8%
Fruit	35%	–	47%	17%	1%
Leaves	15%	32%	–	52%	2%
Flowers	4%	12%	81%	–	4%


### Fungal Interaction Within and Between Plant Organ and Atmospheric Communities

**FIGURE 3:**
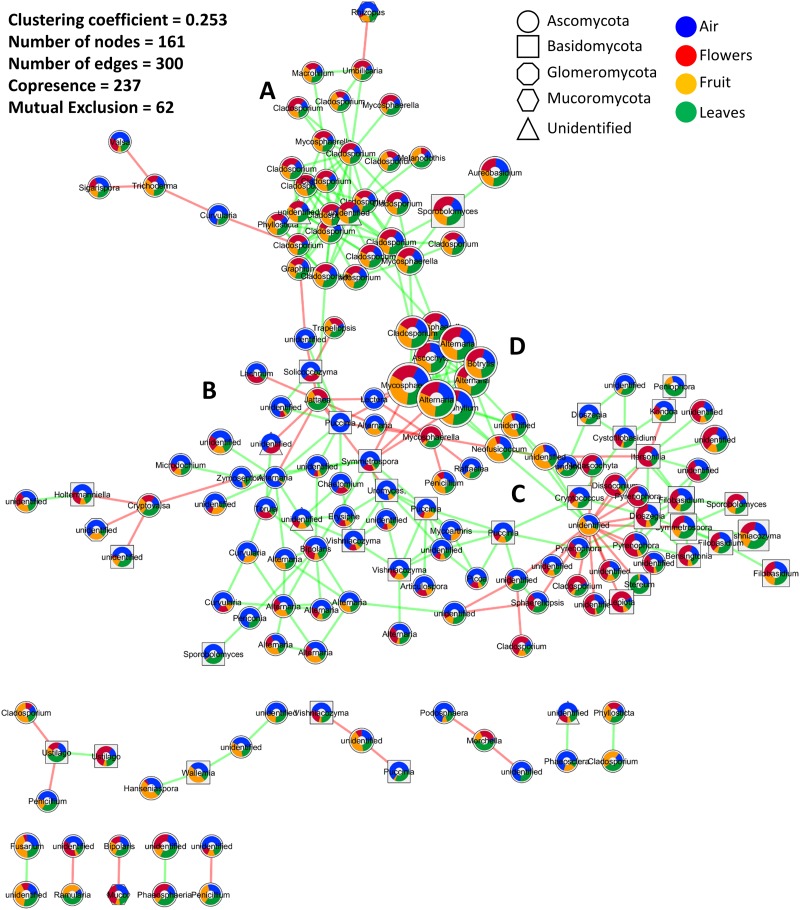
Microbial association network showing interactions (co-occurrence and mutual exclusion) represented by green and red links, respectively. The size of the nods indicates the average relative abundance of OTUs across all investigated samples, while colored pie charts embedded inside the nodes show the relative distribution of each OTU in different samples (air, flowers, fruit, and leaves). Node shapes are used to differentiate fungal phyla. **(A–D)** are used to indicate the four sub-clusters that appeared to be influenced by the average relative abundance distribution of the interacting OTUs in the investigated samples. Interactions were calculated by CoNet, clustered using Connected Components algorithm as implemented in clusterMaker2, and visualized in Cytoscape 3.6.

The co-occurrence and mutual exclusion of specific fungal OTUs were analyzed taking their origin into consideration, i.e., flowers, fruit, leaves, and/or air. The resulting network, after statistical calculations and removal of unstable edges/links, was characterized by 161 nodes (OTUs) linked with 300 edges, and a clustering coefficient of 0.253 (Figure 3). Overall, the interactions between fungal phylotypes were characterized by a higher number of co-occurrences (237) compared to mutual exclusions (62). Highly connected OTUs included taxa of the genus *Cladosporium, Alternaria, Stemphylium, Mycosphaerella*, and an unidentified genus in the order *Pleosporales* (Figure 3). The latter unidentified genus had the highest number of interactions (20) and also the highest number of mutual exclusion relationships (17).

Twelve clusters with an average size of 13.417 were identified using a simple division based on connectivity (Figure 3). A single cluster contained the most interacting OTUs while the other clusters contained 2, 3, or 4 OTUs. Within the main cluster, four sub-clusters appeared to be influenced by the average relative abundance distribution of the interacting OTUs in the investigated samples. These sub-clusters consisted of OTUs mainly associated with plant tissue (sub-cluster A), air samples (sub-cluster B), or evenly distributed among the investigated samples (sub-clusters C and D). Sub-cluster D was similar to sub-cluster A but contained OTUs with the highest abundances. Interactions mainly involved taxa in the phylum *Ascomycota* followed by *Basidiomycota*, unidentified fungi, *Mucoromycota*, and *Glomeromycota*. *Basidiomycota* seemed especially abundant in sub-cluster C. Taxa that were more prevalent in one organ tended to be highly interactive, creating subgroups with fewer connections to other subgroups. This was particularly evident in the air and flowers communities. Furthermore, the majority, if not all, of the negative interactions occurred between taxa from two different subgroups while within-subgroup interactions were more highly characterized by positive interactions.

### Community Composition

The identified OTUs were assigned to 14 fungal phyla, 51 classes, and 1,032 genera. Overall, *Ascomycota* (80.60%), *Basidiomycota* (16.40%), an unidentified phylum (1.60%), *Glomeromycota* (0.5%), and *Mucoromycota* (0.3%) accounted for 99.3% of the total detected taxa (Supplementary Figure S1). At the genus level, *Alternaria, Cladosporium, Mycosphaerella*, unidentified *Pleosporales*, unidentified *Ascomycota, Stemphylium, Aspergillus, Penicillium, Sporobolomyces, Vishniacozyma*, and *Ascochyta* represented over 50% of the total detected fungal genera (Supplementary Figure S2). Beta diversity (Bray–Curtis dissimilarity metric) analyses indicated that air, leaf, and flowers/fruit samples across all sampling times were characterized by significantly different fungal communities (Table 2). In contrast, alpha diversity, evaluated using the Shannon index, varied significantly between atmospheric samples and plant organs but not between samples collected from different plant organs (Table 2). These results were consistent in both vineyards.

**Table 2:** Comparisons between grape organs (flowers, fruits, and leaves) and air samples regardless of the sampling time using alpha (Shannon index) and beta diversity (Bray–Curtis metric).

	Flower		Fruit		Leaves	
			
	Shannon	ANOSIM	Shannon	ANOSIM	Shannon	ANOSIM
Fruit	0.511	0.008 (0.333)				
Leaves	0.743	0.415 (0.017)	0.571	0.002 (0.184)		
Air	0.004	0.001 (0.653)	0.003	0.001 (0.688)	0.006	0.001 (0.560)


*p-values are the results of Student *t*-test method for alpha diversity and ANOSIM test for beta diversity with *R* values between parentheses.*

**FIGURE 4:**
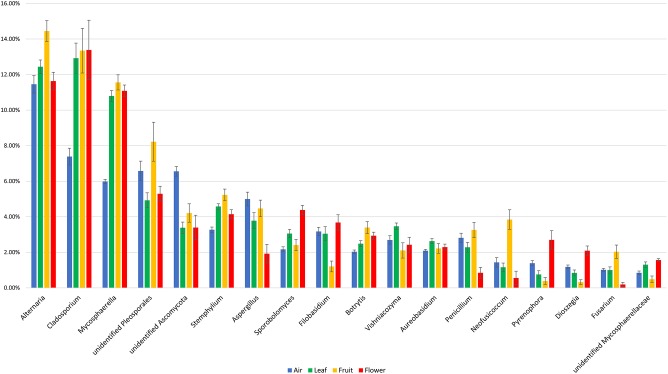
Fungal taxa with significant temporal variations and a relative abundance of at least taxa 1% in different investigated samples (air, fruit, flowers, and leaves). The statistical comparison was done using a non-parametric Kruskal–Wallis test, and the *p* values were calculated through 1000 permutation and corrected using FDR method (Supplementary Table S2).

The majority of fungal genera characterized by a ≥1% relative abundance was detected in all of the investigated sample types. The fungal community composition and relative abundance of different genera, however, varied significantly between the investigated sample types (Figure 4). Some taxa distinguished atmospheric samples from plant organs, having either a significantly lower (*Cladosporium* and *Mycosphaerella*) or higher (unidentified *Ascomycota*) relative abundance. In contrast, genera such as *Alternaria*, unidentified *Pleosporales, Neofusicoccum*, and *Fusarium* were more abundant in fruits than in the other sample types, while *Sporobolomyces, Pyrenophora*, and *Dioszegia* dominated in flowers. A complete list of the fungal genera that significantly differed in their relative abundance between the investigated sample types is presented in Supplementary Table S2.

## Discussion

The present study demonstrated that the atmosphere surrounding the grape phyllosphere has a significantly higher level of fungal diversity than the grape phyllosphere composed of flowers, leaves, and fruits. The high level of fungal diversity was unexpected considering the low availability of nutrients, which is regarded as a key factor in determining microbial diversity in any specific environment (Waldrop et al., 2006; Cline et al., 2018). The data suggest that other factors, such as air currents and the attributes of the local physical environment have more influence on determining atmospheric fungal diversity than nutrient availability.

Several studies have shown that airborne microorganisms are influenced by and may originate from nearby sources such as soil, water, and vegetation (Cho et al., 2006; Behzad et al., 2015). In agreement with these studies, our data indicated that air samples shared a high number of phylotypes (5536 OTUs, 40.3%) with plant organs, and actually a fraction higher than those shared between organs within the same plant.

The Bayesian approach used to estimate the source of the fungal communities also confirmed that a large portion (92%) of the atmospheric fungal community originated from the local plants (grapevines). The same analysis indicated that a smaller percentage (4 to 35%) of the plant-associated fungi originated from the atmosphere, whereas the plant was the major source of its own microbiota. These results, in addition to confirming the role of vegetation in determining the atmospheric microbiome, have important implications on the role of the atmosphere in modulating and mediating the movement of taxa within the plant microbiome. It appears that the plant recruits phyllosphere microorganisms from the atmosphere, which is analogous to the process that occurs between the soil and rhizosphere. Thus, the study demonstrated that the atmosphere serves as a source of recruitment for the fungal communities inhabiting leaves, flowers, and fruits. However, since the atmospheric microbiota surrounding vegetation is highly conditioned by the plants, data also indicate that a fraction of the plant microbiota acquired from the air is originally derived from the same plant. This result raises an important question about the mechanisms of movement/dissemination of microorganisms in the plant canopy ecosystem. Although the internal movement of microbial taxa from one organ to another through the plant is a plausible avenue, the current study makes it difficult to exclude a scenario in which external microbial exchange between plant organs can occur through the atmosphere. Additionally, if a plant genotype selects, and at least partially determines, the microbial species present in its phyllosphere (Berg et al., 2014b), the impressive concentration and diversity of plant-related fungal taxa in the air surrounding a plant surface (plant–air interface) could limit the exchange of microorganisms (between air and plant) to those that already make part of the plant microbiota. Moreover, since the fungal taxa shared between the atmosphere and the plant differed according to the organ, organ-specific selection may also occur. These results, in addition to the fact that we used passive spore traps that were placed for only 1 week, reduces the likelihood that the plant fungal community originated from the atmosphere are merely a random deposition. If random deposition had occurred, similar or even higher levels of fungal diversity would be expected in plant organs than in spore traps and the same shared communities would have been observed between the atmospheric samples and the various plant organs.

The study also demonstrated that a portion of the plant community returns to the plant by recruitment from the atmosphere. This portion of microorganisms can play an important role in the conservation of the plant microbiome. The obtained data support this premise in that all of the tested environments (plant organs and air samples) contained a shared community that served as a two-way source/reservoir of microorganisms. For example, the data analysis indicated that 23.7, 35.7, 61.3, and 44.4% of air, fruit, leaf, and flower communities return to these organs by recruitment from the other investigated environments. These shared factions represent a common reservoir/storage of microorganisms that could aid in maintaining and preserving the identity of the microbial signature associated with each organ. In this regard, leaves appear to be the most influential environment, sharing and receiving 61.3% of their communities with/from other environments. In contrast, air samples appeared to be the least influential environment, sharing only 23% of its fungal taxa with the various plant organs. These results, even though surprising, are supported by the well-documented stability of a plant’s microbiome within a species (Laforest-Lapointe et al., 2016, 2017; Liu et al., 2018). The plant–air interface not only plays a role as a reservoir of the taxa making up the plant microbiome, mediating the movement of these microorganisms, but also shelters the plant from invasive species. Some of the observed mutual exclusions between OTUs prevalent on the plant and OTUs prevalent in air samples suggest competition at the niche level, mainly between the plant and atmosphere. These findings also support the idea that microorganisms emitted by the plant into the atmosphere could serve as a shield, protecting the plant itself and the plant’s indigenous microorganisms from alien/invasive species of microorganisms. It is important to note that a considerable number of taxa detected in both air and plant samples were phytopathogens, including *Botrytis, Erysiphe, Eutypa, Phomopsis, Fomitiporia, Phaeomoniella, Phaeoacremonium, Botryosphaeria*, and *Neofusicoccum, Aspergillus, Didymella, Mucor*, and *Rhizopus*. Therefore, understanding the microbial movement between plant organs and the atmosphere, as well as the interactions between microorganisms, presents a new paradigm for the development of disease management strategies.

## Data Availability

The datasets generated during the current study were deposited and are available at the National Center for Biotechnology Information (NCBI), Sequence Read Archive (SRA), under the accession number PRJNA494163 (www.ncbi.nlm.nih.gov/sra/PRJNA494163). Other data generated or analyzed during this study are included in this published article and its additional files.

## Author Contributions

AA and LS conceived and designed the experiments and analyzed the data. AA performed the experiments. LS coordinated the overall study and edited the manuscript. All authors interpreted the results, wrote the manuscript, and read and approved the manuscript.

## Conflict of Interest Statement

The authors declare that the research was conducted in the absence of any commercial or financial relationships that could be construed as a potential conflict of interest.

## Footnotes

1 www.illumina.com

2 https://github.com/biota/sourcetracker2

## Supplementary Material

FIGURE S1 Krona chart showing the relative abundance of the detected phyla, classes, and order across all the investigated samples.

FIGURE S2 Krona chart showing the relative abundance of the detected families, and genera across all the investigated samples.

TABLE S1 Summary of the investigated samples in the current study (air, leaf, flower, and fruit) from different sampling points (June, July, August, September, and October) from table grape vineyards. The table shows the total number of reads and OTUs observed in each sample as well as alpha diversity (Shannon index).

TABLE S2 List of fungal genera that significantly changed between the investigated environments according Kruskal–Wallis test.
