# Supplementary figures and images for: Revealing Cues for Fungal Interplay in the Plant–Air Interface in Vineyards
Source: Front Plant Sci. 2019 Jul 25;10:922. doi: 10.3389/fpls.2019.00922 (PMC6670289; doi:10.3389/fpls.2019.00922)

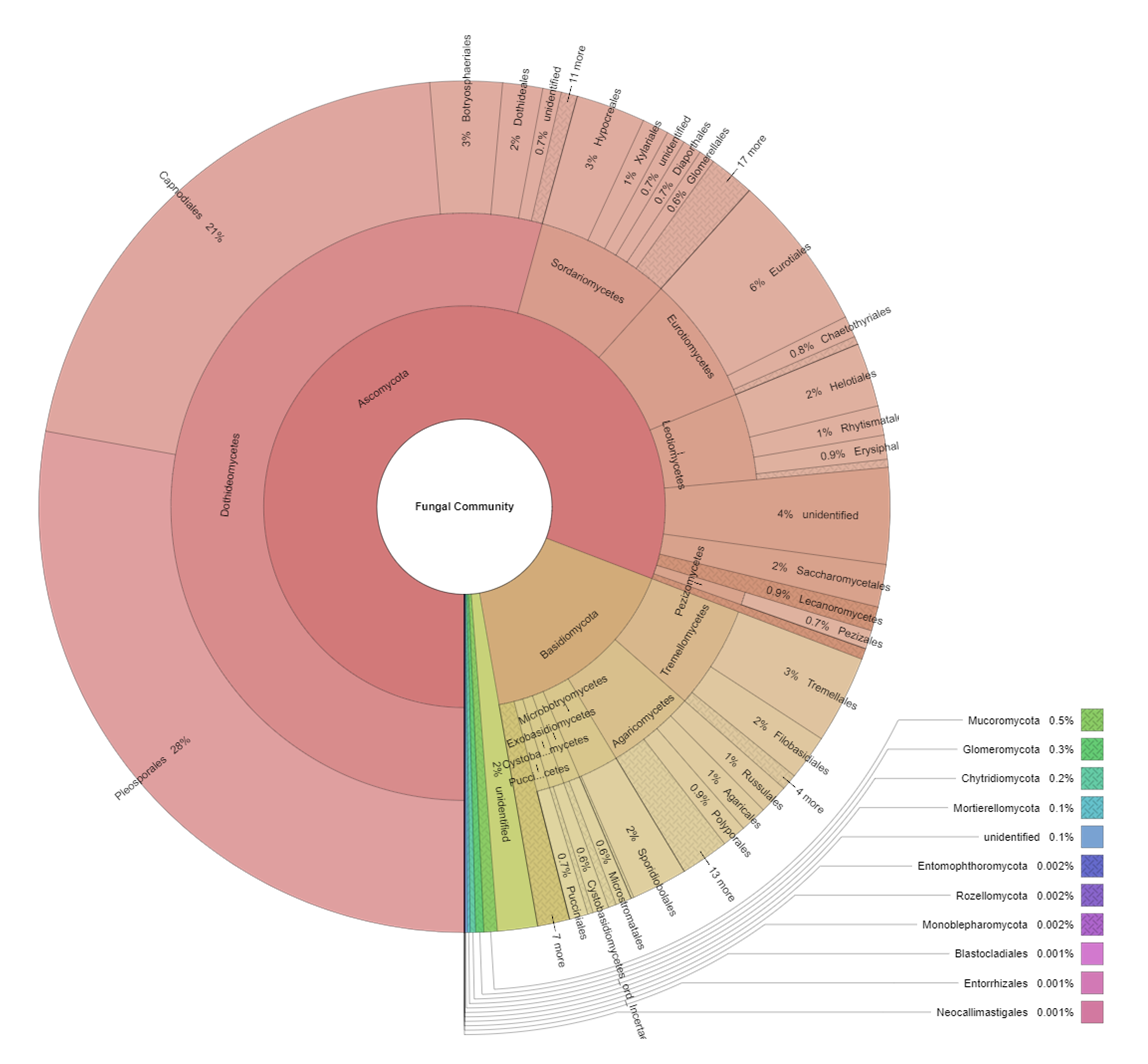

Supplement: FIGURE S1 — Krona chart showing the relative abundance of the detected phyla, classes, and order across all the investigated samples. [file Image_1.TIF]

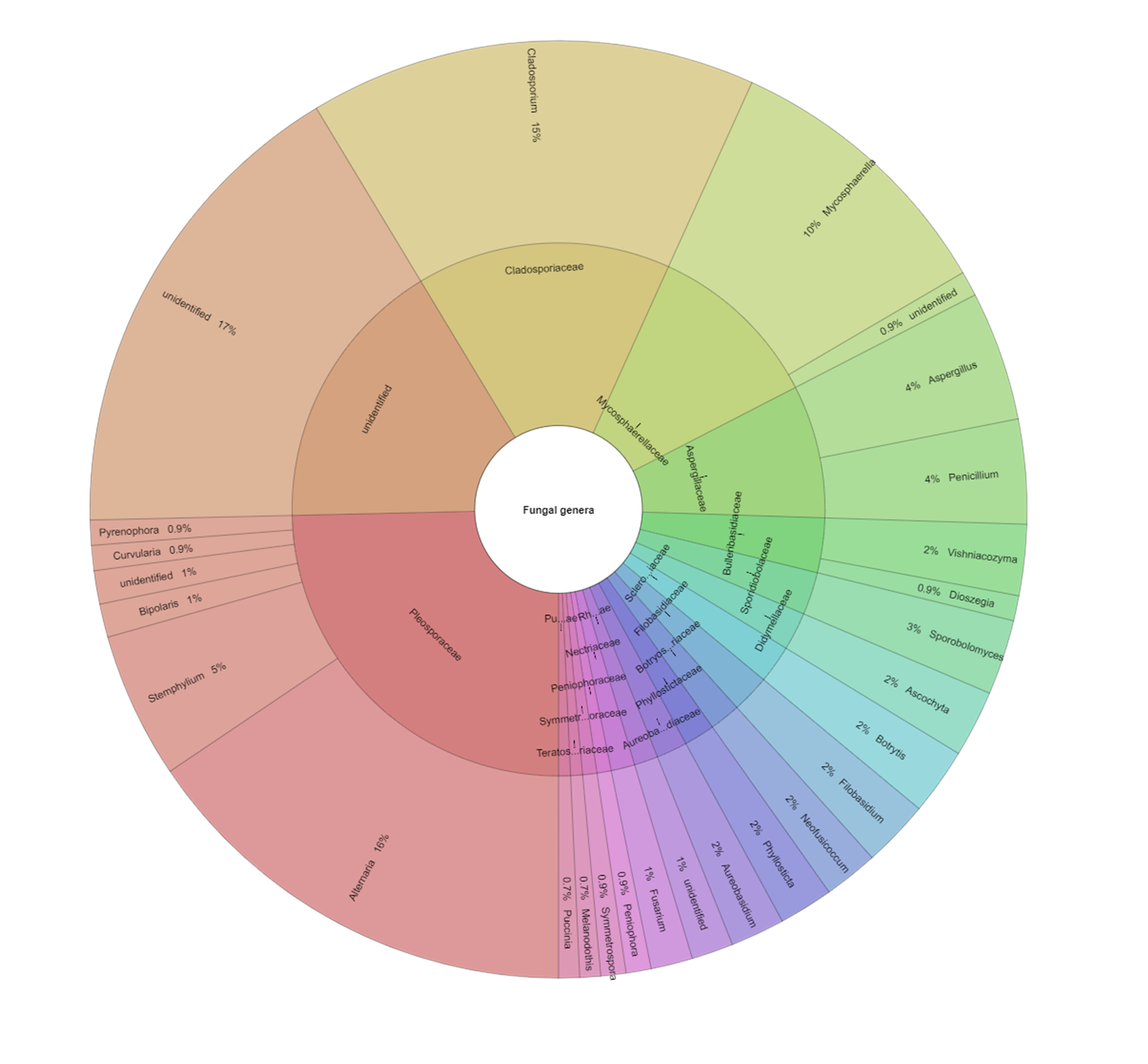

Supplement: FIGURE S2 — Krona chart showing the relative abundance of the detected families, and genera across all the investigated samples. [file Image_2.TIF]
