# Supplementary material for: Revealing Cues for Fungal Interplay in the Plant–Air Interface in Vineyards
Source: Front Plant Sci. 2019 Jul 25;10:922. doi: 10.3389/fpls.2019.00922 (PMC6670289; doi:10.3389/fpls.2019.00922)
Supplement: TABLE S1 — Summary of the investigated samples in the current study (air, leaf, flower, and fruit) from different sampling points (June, July, August, September, and October) from table grape vineyards. The table shows the total number of reads and OTUs observed in each sample as well as alpha diversity (Shannon index). [file Table_1.docx]

Supplementary Table S1

Summary of the investigated samples in the current study (air, leaf, flower, fruit) from different sampling points (June, July, August, September, October) from table grape vineyards. The table shows the total number of reads and OTUs observed in each sample as well as alpha diversity (Shannon index).

| **Vineyard** | **Sampling period** | **Organ** | **Shannon*** | **No. reads** | **No. OTUs** |
| --- | --- | --- | --- | --- | --- |
| **Vineyard** **1** | June | Leaf | 1.95 | 126261 | 965 |
|  | June | Flower | 1.65 | 178958 | 1100 |
|  | June | Air | 3.18 | 291056 | 2026 |
|  | July | Leaf | 1.91 | 10241 | 609 |
|  | July | Fruit | 2.24 | 77393 | 561 |
|  | July | Air | 2.82 | 276312 | 1845 |
|  | August | Leaf | 1.49 | 76978 | 548 |
|  | August | Fruit | 2.39 | 32847 | 278 |
|  | August | Air | 3.01 | 296138 | 1839 |
|  | September | Leaf | 1.57 | 101966 | 733 |
|  | September | Fruit | 2.57 | 99032 | 663 |
|  | September | Air | 3.37 | 289886 | 2230 |
|  | October | Leaf | 1.77 | 540501 | 2632 |
|  | October | Fruit | 1.55 | 348385 | 1730 |
|  | October | Air | 3.17 | 519032 | 2577 |
| **Vineyard** **2** | June | Leaf | 3.16 | 33952 | 569 |
|  | June | Flower | 2.82 | 20297 | 411 |
|  | June | Air | 4.44 | 219325 | 2114 |
|  | July | Leaf | 2.98 | 43553 | 471 |
|  | July | Fruit | 2.84 | 4401 | 105 |
|  | July | Air | 3.35 | 346994 | 2674 |
|  | August | Leaf | 2.89 | 2084 | 140 |
|  | August | Fruit | 3.28 | 5802 | 151 |
|  | August | Air | 3.24 | 216498 | 1468 |
|  | September | Leaf | 2.96 | 6413 | 223 |
|  | September | Fruit | 2.52 | 35993 | 338 |
|  | September | Air | 3.19 | 202634 | 1022 |
|  | October | Leaf | 2.61 | 34401 | 511 |
|  | October | Fruit | 2.56 | 281224 | 1571 |
|  | October | Air | 2.82 | 755484 | 3680 |
