# Supplementary material for: Revealing Cues for Fungal Interplay in the Plant–Air Interface in Vineyards
Source: Front Plant Sci. 2019 Jul 25;10:922. doi: 10.3389/fpls.2019.00922 (PMC6670289; doi:10.3389/fpls.2019.00922)
Supplement: TABLE S2 — List of fungal genera that significantly changed between the investigated environments according Kruskal–Wallis test. [file Table_2.docx]

**Supplementary Table S2**

List of fungal genera that significantly changed between the investigated environments according Kruskal-Wallis test.

| OTU | FDR P | Air | Leaf | Fruit | Flower |
| --- | --- | --- | --- | --- | --- |
| *Alternaria* | 0.001840326 | 11.46% | 12.44% | 14.45% | 11.64% |
| *Cladosporium* | 8.43E-05 | 7.39% | 12.92% | 13.35% | 13.38% |
| *Mycosphaerella* | 5.26E-11 | 5.98% | 10.80% | 11.56% | 11.08% |
| unidentified *Pleosporales* | 0.034090897 | 6.58% | 4.93% | 8.22% | 5.30% |
| unidentified *Ascomycota* | 1.72E-07 | 6.56% | 3.38% | 4.21% | 3.39% |
| *Stemphylium* | 2.49E-07 | 3.27% | 4.58% | 5.23% | 4.14% |
| *Aspergillus* | 0.014805944 | 5.00% | 3.78% | 4.47% | 1.92% |
| *Sporobolomyces* | 0.000416648 | 2.17% | 3.06% | 2.41% | 4.38% |
| *Filobasidium* | 0.000124117 | 3.17% | 3.05% | 1.20% | 3.68% |
| *Botrytis* | 0.000604468 | 2.03% | 2.49% | 3.40% | 2.93% |
| *Vishniacozyma* | 0.009199212 | 2.70% | 3.47% | 2.11% | 2.42% |
| *Aureobasidium* | 0.040556296 | 2.09% | 2.63% | 2.22% | 2.30% |
| *Penicillium* | 0.009835286 | 2.81% | 2.28% | 3.26% | 0.85% |
| *Neofusicoccum* | 0.000103252 | 1.43% | 1.16% | 3.84% | 0.56% |
| *Pyrenophora* | 1.05E-05 | 1.39% | 0.76% | 0.39% | 2.70% |
| *Dioszegia* | 4.86E-05 | 1.18% | 0.84% | 0.32% | 2.09% |
| *Fusarium* | 0.002687411 | 1.01% | 1.00% | 2.03% | 0.19% |
| unidentified *Mycosphaerellaceae* | 0.001349588 | 0.85% | 1.31% | 0.49% | 1.57% |
| unidentified *Pleosporaceae* | 2.85E-06 | 1.53% | 0.93% | 0.23% | 1.12% |
| *Symmetrospora* | 0.007108276 | 0.83% | 1.19% | 0.51% | 1.10% |
| unidentified *Sordariomycetes* | 5.14E-05 | 1.51% | 0.39% | 0.73% | 0.56% |
| *Bipolaris* | 1.16E-07 | 1.21% | 0.45% | 0.16% | 0.78% |
| *Dissoconium* | 1.96E-06 | 0.34% | 0.20% | 0.10% | 1.72% |
| *Gibberella* | 0.000723247 | 0.83% | 0.63% | 0.22% | 0.59% |
| *Phaeosphaeria* | 0.000708238 | 0.35% | 0.76% | 0.12% | 0.89% |
| *Jattaea* | 2.49E-07 | 0.04% | 0.81% | 0.41% | 0.58% |
| *Lepiota* | 0.001414288 | 0.11% | 0.21% | 0.20% | 1.30% |
| *Microstroma* | 0.002450202 | 0.73% | 0.65% | 0.32% | 0.11% |
| unidentified *Rhytismataceae* | 0.013624101 | 0.78% | 0.41% | 0.21% | 0.41% |
| *Cercospora* | 2.71E-06 | 0.64% | 0.15% | 0.12% | 0.71% |
| *Itersonilia* | 2.85E-06 | 0.21% | 0.24% | 0.00% | 1.14% |
| *Bensingtonia* | 0.000224204 | 0.30% | 0.25% | 0.10% | 0.90% |
| *Cryptococcus* | 0.005508485 | 0.59% | 0.42% | 0.17% | 0.36% |
| unidentified *Phaeosphaeriaceae* | 0.000252618 | 0.54% | 0.27% | 0.09% | 0.59% |
| *Septoriella* | 0.002634324 | 0.31% | 0.24% | 0.21% | 0.57% |
| *Curvularia* | 2.47E-06 | 0.94% | 0.20% | 0.13% | 0.00% |
| *Golovinomyces* | 0.004655145 | 0.31% | 0.38% | 0.08% | 0.39% |
| *Neoascochyta* | 0.009271259 | 0.16% | 0.37% | 0.00% | 0.58% |
| unidentified *Dothideomycetes* | 0.000417986 | 0.34% | 0.16% | 0.06% | 0.53% |
| *Spegazzinia* | 0.000108078 | 0.42% | 0.24% | 0.25% | 0.18% |
| *Periconia* | 2.81E-07 | 0.71% | 0.33% | 0.03% | 0.00% |
| *Holtermanniella* | 0.000102347 | 0.47% | 0.20% | 0.13% | 0.26% |
| *Plectosphaerella* | 8.74E-05 | 0.48% | 0.27% | 0.22% | 0.00% |
| *Wallemia* | 0.000105985 | 0.35% | 0.12% | 0.48% | 0.00% |
| *Sphaerellopsis* | 0.01455197 | 0.33% | 0.31% | 0.01% | 0.29% |
| unidentified *Tremellomycetes* | 0.00563119 | 0.38% | 0.31% | 0.17% | 0.04% |
| *Colletotrichum* | 0.000452047 | 0.47% | 0.10% | 0.30% | 0.03% |
| *Puccinia* | 0.000150657 | 0.46% | 0.16% | 0.15% | 0.12% |
| *Cystofilobasidium* | 0.000180131 | 0.39% | 0.22% | 0.03% | 0.25% |
| *Microdochium* | 0.009199212 | 0.41% | 0.22% | 0.10% | 0.13% |
| *Rhizopus* | 0.03842061 | 0.46% | 0.17% | 0.17% | 0.04% |
| *Vuilleminia* | 0.000150657 | 0.33% | 0.21% | 0.12% | 0.18% |
| *Sigarispora* | 0.002998183 | 0.27% | 0.24% | 0.12% | 0.20% |
| *Bullera* | 0.000102347 | 0.37% | 0.18% | 0.03% | 0.23% |
| *Kondoa* | 0.000224204 | 0.32% | 0.28% | 0.06% | 0.12% |
| *Erysiphe* | 4.71E-06 | 0.49% | 0.09% | 0.08% | 0.09% |
| *Sarocladium* | 0.02333266 | 0.28% | 0.23% | 0.05% | 0.13% |
| unidentified *GS17* | 0.000604468 | 0.40% | 0.19% | 0.09% | 0.00% |
| *Uwebraunia* | 0.001840326 | 0.27% | 0.31% | 0.09% | 0.00% |
| *Latorua* | 0.000497814 | 0.34% | 0.27% | 0.05% | 0.00% |
| *Torula* | 2.49E-07 | 0.39% | 0.06% | 0.01% | 0.18% |
| *Paraconiothyrium* | 2.04E-05 | 0.28% | 0.25% | 0.09% | 0.00% |
| *Devriesia* | 0.00736748 | 0.22% | 0.28% | 0.13% | 0.00% |
| *Raffaelea* | 2.04E-05 | 0.37% | 0.20% | 0.06% | 0.00% |
| *Erythrobasidium* | 0.000249551 | 0.29% | 0.21% | 0.10% | 0.00% |
| *Setosphaeria* | 0.012938671 | 0.38% | 0.16% | 0.03% | 0.00% |
| *Nigrospora* | 0.00032426 | 0.31% | 0.19% | 0.04% | 0.00% |
| *Zymoseptoria* | 1.36E-06 | 0.42% | 0.09% | 0.02% | 0.00% |
| *Neoidriella* | 2.04E-05 | 0.32% | 0.15% | 0.05% | 0.00% |
